# Downregulation of long noncoding RNA MEG3 is associated with poor prognosis and promoter hypermethylation in cervical cancer

**DOI:** 10.1186/s13046-016-0472-2

**Published:** 2017-01-05

**Authors:** Jun Zhang, Zhongqiu Lin, Yali Gao, Tingting Yao

**Affiliations:** 1 Department of Obstetrics and Gynecology, The Second Clinical Medical College (Shenzhen People’s Hospital), Jinan University, Shenzhen, 518020 People’s Republic of China; 2 Department of Gynecological Oncology, Sun Yat-sen Memorial Hospital, Sun Yat-sen University, Guangzhou, 510120 People’s Republic of China; 3 Department of Ophthalmology, The Second Clinical Medical College (Shenzhen People’s Hospital), Jinan University, Shenzhen, 518020 People’s Republic of China

**Keywords:** Cervical cancer, Diagnosis, Long noncoding RNA, *MEG3*, Methylation, Prognosis

## Abstract

**Background:**

Our previous study reported that *MEG3* is an important tumor suppressor gene that is inactivated in cervical cancer. However, the diagnostic and prognostic values of *MEG3,* as well as the molecular mechanism of *MEG3* inactivation in cervical cancer, remain unclear. In this study, we aimed to further elucidate the role and potential inactivation mechanism of *MEG3* in cervical cancer.

**Methods:**

ROC curve and Cox regression analyses were used to assess the diagnostic and prognostic value of *MEG3* in patients with cervical cancer. The methylation status of the MEG3 promoter in cervical cancer tissue samples was tested using methylation-specific PCR. Furthermore, we altered the methylation status of the *MEG3* promoter in two cervical cancer cell lines (HeLa and CaSki) using a DNA methylation transfer enzyme inhibitor (5-Aza-CdR), to investigate whether promoter hypermethylation is a potential cause of *MEG3* inactivation. Finally, we used CCK-8 and colony formation assays to evaluate the cell proliferation ability of HeLa and CaSki cells that had been treated with 5-aza-CdR, to investigate whether downregulation of *MEG3* caused by promoter hypermethylation had biological effects.

**Results:**

ROC curve analysis indicated that *MEG3* status showed sufficient sensitivity and specificity for prediction of tumor size and lymph node metastasis in patients with cervical cancer. In addition, our follow-up data showed that low *MEG3* expression was correlated with recurrence and short overall survival. Moreover, hypermethylation of the *MEG3* promoter was observed in most cervical cancer tissue samples, and demethylation of the *MEG3* promoter led to re-expression of *MEG3* and inhibited proliferation of HeLa and CaSki cells.

**Conclusions:**

*MEG3* is a powerful tool for diagnosis and prognosis of patients with cervical cancer, and low expression of *MEG3* is likely to be related to promoter hypermethylation in cervical cancer.

**Electronic supplementary material:**

The online version of this article (doi:10.1186/s13046-016-0472-2) contains supplementary material, which is available to authorized users.

## Background

Cervical cancer is one of the most commonly diagnosed cancers among women worldwide, accounting for over 500,000 new cases and 260,000 cases of death annually [1]. Although the incidence and mortality rates of cervical cancer have declined over the past 30 years, the 5-year survival rate of advanced-stage patients has remained below 40% [2, 3]. Therefore, it is important to explore the molecular mechanisms of cervical cancer in order to identify effective prognostic markers and design improved therapeutic strategies.

Long noncoding RNA (lncRNA) is usually defined as an RNA molecule that is longer than 200 nucleotides and lacks significant protein-coding capacity [4]. Studies have reported that lncRNAs regulate various biological processes such as gene expression, transcription, and cellular proliferation, among others [5, 6]. More importantly, abnormal expression of lncRNAs was also found to be involved in metastasis, recurrence, and prognosis of various human cancers [7]. Maternally expressed gene 3 (*MEG3*), a lncRNA that has attracted much research interest, is aberrantly expressed in several human cancers including gastric cancer [8, 9], colorectal cancer [10], retinoblastoma [11], and ovarian cancer [12] according to recent studies. Moreover, our previous study showed that *MEG3* is associated with the progression of cervical cancer via regulation of cell proliferation and apoptosis [13]. However, the diagnostic and prognostic value of *MEG3* for cervical cancer remains unknown.

In addition to inducing abnormal expression of tumor suppressor genes and oncogenes, epigenetic alterations play an important role in the development of cancers. Many studies have confirmed that hypermethylation of gene promoter regions can directly cause a decrease in gene expression levels [14]. Hypermethylation of the promoter region of tumor suppressor genes, including genes that encode lncRNAs, is a common cause of cancer [15]. However, no reports have been published to date on the relationship between promoter methylation and *MEG3* expression in cervical cancer.

In this study, we investigated the relationship between *MEG3* and cervical cancer based on the results of our previous study. We first performed ROC curve and Cox regression analyses to determine the clinical value of *MEG3* in patients with cervical cancer. Then we identified the epigenetic regulation of *MEG3* expression in cervical cancer tissues and cells. These results deepen our understanding of the role of *MEG3* in cervical cancer.

## Methods

### Patient samples

Seventy-two cervical cancer tissue and its corresponding normal tissues were obtained from patients admitted to our hospital for surgery from April 2012 to March 2013. The specimens were immediately placed in liquid nitrogen after surgical resection. All the patients were newly diagnosed as cervical cancer without receiving any treatment. The samples which considered as cervical cancer or normal tissues were determined by pathologic examination. Written consent was obtained from each patient before tissue collection. The protocol was approved by the Institutional Research Ethics Committee of our hospital. The patients’ clinico-pathological characteristics are summarized in Additional file 1: Table S1.

### Cell lines and culture

Cervical cancer cell lines HeLa and CaSki were purchased from the Institute of Biochemistry and Cell Biology at the Chinese Academy of Sciences (Shanghai, China). HeLa cells were maintained in DMEM (Gibco, Gaithersburg, MD, USA) supplemented with 10% fetal bovine serum (FBS, Gibco) and CaSki cells were cultured in RPMI 1640 medium (Gibco) with 10% FBS at 37 °C in a humidified incubator of 5% CO_2_.

### Transfection of siRNA

SiRNA for this study was the same as our previous study. Briefly, the siRNA for knockdown *MEG3* (si-*MEG3*) and its negative control (si-NC) were designed and synthesized by GenePharma (Shanghai, China). SiRNA was transfected using Lipofectamine 2000 (Invitrogen, Carlsbad, California, USA) according to the manufacturer’s protocol. 48 h after transfection, cells were harvested and subjected to qRT-PCR analyses and functional assays.

### RNA isolation and qRT-PCR

Total RNA was isolated from tissues and cells using Trizol reagent (TaKaRa, Otsu, Japan) according to the manufacturer’s instructions. Concentration and purification of RNA were determined by measuring its optical density using NanoDrop 2000 Spectrophotometer (1.8 < A260/280 < 2.0, Thermo Scientific Wilmington, DE, USA). Reverse transcription was carried out using PrimeScriptTM RT reagent Kit following the manufacturer’s protocol (TaKaRa). QRT-PCR was performed using a SYBR Premix Ex Taq II kit (TaKaRa). Briefly, reactions were loaded into a 96-well plate in duplicate and firstly incubated at 95 °C for 5 min, followed by 40 cycles of denaturation at 95 °C for 30 s, 1 min of annealing at 60 °C and extension at 60 °C for 1 min on CFX96 Real-Time PCR Detection System (Bio-Rad, Hercules, California, US). The β-Actin was chosen as the endogenous normalizer and the 2^−ΔΔCt^ method was used to determine relative expression of *MEG3*. The primers used for qRT-PCR in this study are the same as our previous study.

### Methylation specific PCR for promoter of *MEG3*

First, DNA from cells and tissues was extracted using the QIAmp DNA Mini kit (Qiagen, German), according to the manufacturer’s recommendations. Next, we used the EpiTect Bisulfite kit (Qiagen) for bisulfate conversion following the product manual. At last, Methylation specific PCR (MSP) was performed on Bisulfite-treated DNA using EpiTect MSP kit (Qiagen). The primers of *MEG3* promoter which specific for methylated and unmethylated were as follows: the methylated pair (M) was 5′-GTT AGT AAT CGG GTT TGT CGG C (forward) and 5′-AAT CAT AAC TCC GAA CAC CCG CG (reverse); the unmethylated pair (U) was 5′-GAG GAT GGT TAG TTA TTG GGG T (forward) and 5′-CCA CCA TAA CCA ACA CCC TAT AAT CAC A (reverse). The PCR reaction was conducted in DNA Engine Tetrad 2 Peltier Thermal Cycler (Bio-Rad) under the following conditions: 95 °C 15 min; 94 °C 30s, 70 °C 30s, 72 °C 30s, 5 cycles; 94 °C 30s, 65 °C 30s, 72 °C 30s, 5 cycles; 94 °C 30s, 60 °C 30s, 72 °C 30s, 30 cycles; 72 °C 7 min. The PCR products were identified by electrophoresis through 2.5% agarose gel, stained with ethidium bromide, visualized under UV and compared by densitometry.

### Treatment with 5-Aza-CdR and DZNep

The DNA demethylating agent 5-aza-2-deoxy-cytidine (5-Aza-CdR) was obtained from Sigma-Aldrich (MO, USA) and diluted in dimethyl sulfoxide (DMSO, MP Biomedicals, California, USA). Total 1 × 10^5^ cells were seeded in six-well culture plate and treated with 0, 5, 10 μmol/L 5-aza-CdR for next 5 days. The medium was replaced with the same concentration of 5-Aza-CdR every day. Then cells were collected and prepared for qRT-PCR and MSP to measure the expression of MEG3 and the methylation status of the MEG3 promoter respectively. For CCK-8 assay, we treated cells with 10 μmol/L 5-aza-CdR for 5 days, following transfection of si-*MEG3* and compared proliferation ability with cells which were treated with 5-Aza-CdR only.

3-Deazaneplanocin A (DZNep), an inhibitor of the histone methyltransferase EZH2, was purchased from Sigma-Aldrich (MO, USA). The cells were seeded at 1 × 10^5^ cells per well and treated with DZNep at 0, 1 and 5 μmol/L for 5 days. After that, cells were harvested for qRT-PCR and Western Blot.

### CCK-8 assay

Cell proliferation ability was evaluated using Cell Counting Kit-8 (CCK-8, Dojindo, Kumamoto, Japan) following the manufacturer’s instructions. Briefly, Hela and Caski cells were harvested 48 h post-transfection before seeding into a 96-well plate at a density of 3x10^3^ cells per well. After 6 h of incubation, 10 μl CCK-8 was added to each well at 0, 24, 48 or 72 h time point. Cells were incubated for 1.5 h at 37 °C and the absorbance was read at 450 nm using SoftMax pro5 Microplate Reader (Molecular Devices, California, USA).

### Colony formation assay

For Colony formation assay, 1x10^3^ cells per well were seeded into the 6-well plates and cultured for 7 days in complete growth media. Afterwards, the adherent cells were washed with PBS, fixed with 10% paraformaldehyde for 10 min, stained with 1% Crystal violet for 5 min, photographed and counted.

### Western blot

The cells were lysed with RIPA (Thermo Scientific, USA) supplemented with protease inhibitors (Roche, Switzerland) according to the manufactures protocol. Fifty micrograms of proteins were separated by SDS-PAGE and transferred to PVDF membranes (Roche, Switzerland). Afterwards, the membranes were blocked and incubated with rabbit anti-human EZH2 antibody (1:1,000; CST, USA) at 4 °C overnight. Then membranes were washed with TBST and probed with HRP Goat-anti-Rabbit (1:2000; Santa Cruz Biotechnology) at room temperature for 2 h. At last, the proteins were measured semiquantitatively with ECL (Thermo Scientific) and normalized to the expression of β-Actin (1:1,000; CST, USA).

### Statistical analysis

Each experiment was performed in triplicate and the results were represented as mean ± SEM. The SPSS software (version 11.0, SPSS Inc, USA) was used for the statistical analyses. Differences between groups were compared using the Student’s t test or LSD test for continuous variables and the Pearson’s chi-square test for qualitative variables. Statistical significance was considered at *P* ≤ 0.05.

## Results

### Diagnostic value of *MEG3* for patients with cervical cancer

**Fig. 1:**
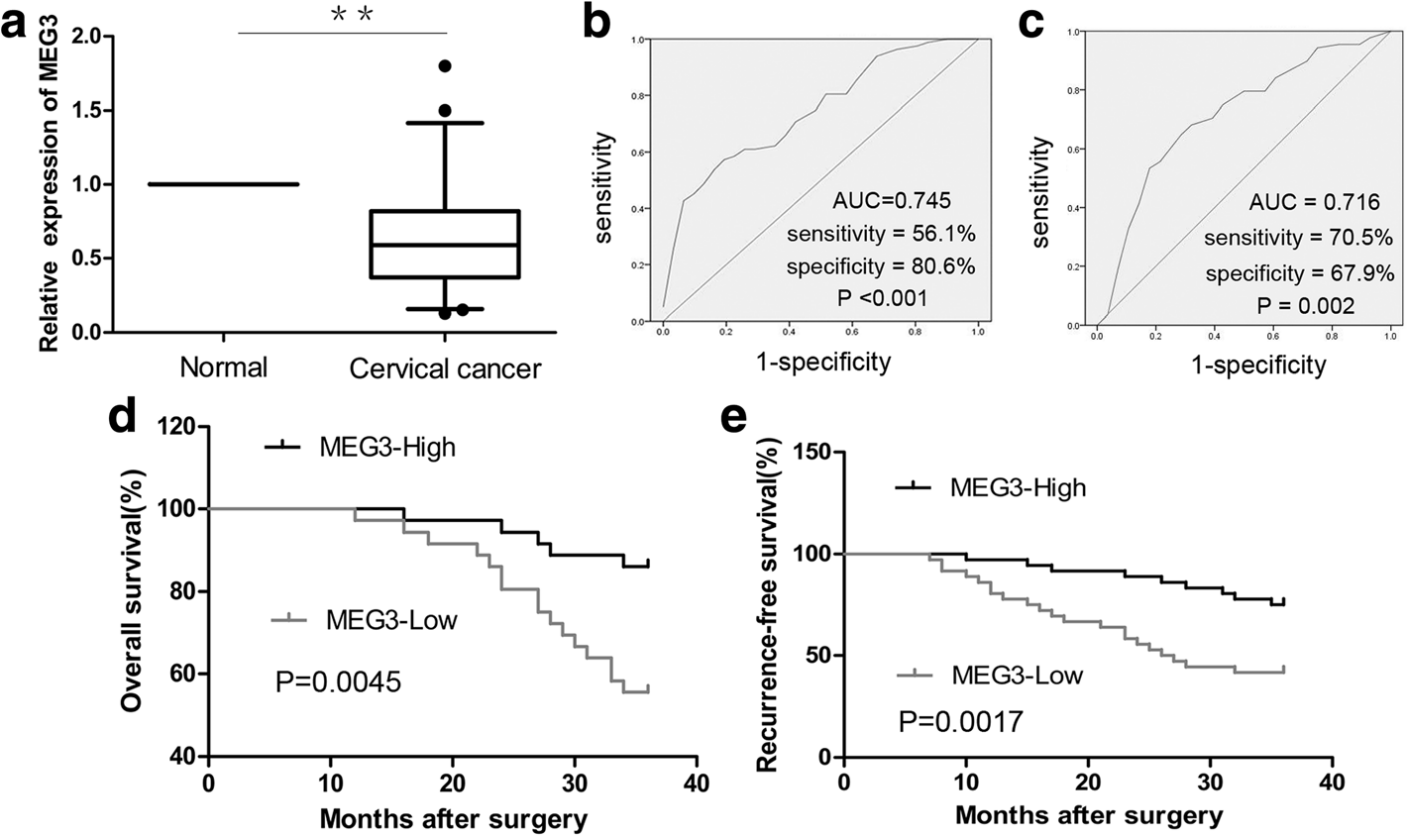
The diagnostic and prognostic value of *MEG3* in cervical cancer. **a** Relative expression of *MEG3* in cervical cancer tissues (*n* = 72) and corresponding normal tissues (*n* = 72). ROC curve of *MEG3* expression predicts the tumor size (**b**) and presence of lymph nodes metastasis (**c**) in terms of sensitivity and specificity. **d**, **e** Patients in *MEG3*-Low group (*n* = 36) had significantly shorter recurrence-free and overall survival than those in *MEG3*-High group (*n* = 36). *P* value was calculated by Log-rank test.^**^ *P* < 0.01

The results of qRT-PCR analysis showed that *MEG3* expression was significantly lower in cervical cancer tissues compared to corresponding normal tissues, consistent with our previous study (Fig. 1a). Because *MEG3* was shown to be significantly associated with tumor size and lymph node metastasis in our previous study, we conducted receiver operating characteristic (ROC) curve analysis to test its diagnostic value. The results showed that *MEG3* could be a candidate to discriminate between tumors < 4 cm and tumors ≥ 4 cm, with an AUC (area under the ROC curve) of 0.745, a sensitivity of 56.1%, and a specificity of 80.6% at a cut-off value of 0.705 (*P* < 0.001, Fig. 1b). ROC curve analysis also revealed that *MEG3* could serve as a biomarker for lymph node metastasis, with an AUC of 0.716. At a cut-off value of 0.475, the sensitivity and the specificity were 70.5% and 67.9%, respectively (*P* = 0.002, Fig. 1c).

**Fig. 2:**
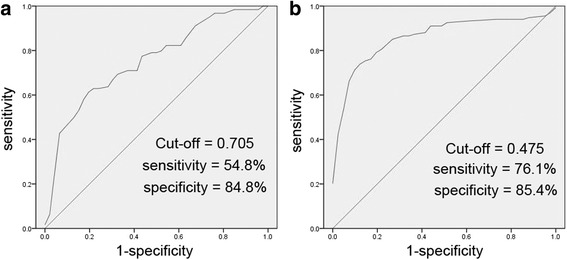
*MEG3* cut-off values for tumor size (**a**) and presence of lymph nodes metastasis (**b**) setting before were tested by other 108 patients with cervical cancer from our previous study. The AUC was 0.753 (*P* < 0.001, **a**) and 0.862 (*P* < 0.001, **b**), respectively

In addition, we tested the ROC model above using data collected from 108 patients with cervical cancer in our previous study. The AUCs for tumor size and lymph node metastasis were 0.753 and 0.862, respectively (*P* < 0.001, Fig. 2). Furthermore, at a cut-off value of 0.705, the sensitivity and specificity for diagnosis of tumor size (<4 cm or ≥ 4 cm) were 54.8% and 84.8%, respectively (Fig. 2a). When the cut-off value was 0.475, the sensitivity and specificity for prediction of lymph node metastasis were 76.1% and 85.4%, respectively (Fig. 2b).

### Relationship between *MEG3* expression and prognosis

To further evaluate whether *MEG3* expression is linked to survival, patients with cervical cancer (*n* = 72) were classified into the *MEG3*-Low (*n* = 36) and *MEG3*-High groups (*n* = 36) according to the median value of *MEG3* expression. Kaplan–Meier analysis showed that patients in the *MEG3*-Low group had significantly shorter recurrence-free and overall survival than those in the *MEG3*-High group (*P* < 0.01, Fig. 1d, e).

Furthermore, we tested the predictive value of *MEG3* for relevant clinical and pathological parameters, including tumor size, FIGO stage, lymph node metastasis, HR-HPV infection, age, menopause, histology, depth of invasion, differentiation, and lymphatic vascular space invasion (LVSI). Univariate analysis showed that *MEG3* expression, differentiation, FIGO stage, and lymph node metastasis were all prognostic factors for recurrence-free survival in patients with cervical cancer (Table 1). In a multivariate analysis based on the Cox proportional hazards regression model, only *MEG3* expression, FIGO stage, and lymph node metastasis were found to be independent prognostic markers for recurrence-free survival (Table 1).

**Table 1:** Univariate and Multivariate analyses for recurrence-free survival

Risk factors	Univariate analysis			Multivariate analysis		
	HR	*P* value	95% CI	HR	*P* value	95% CI
*MEG3* expression	0.111	0.002	0.027 ∼ 0.451	0.159	0.040	0.028 ∼ 0.919
FIGO stage, (I, II)	3.687	<0.001	1.780 ∼ 7.634	3.033	0.005	1.400 ∼ 6.571
Lymph nodes metastasis (Negative, Positive)	2.095	0.043	1.023 ∼ 4.291	2.259	0.048	1.007 ∼ 5.070
Differentiation (Well/Moderately, Poorly)	2.134	0.040	1.036 ∼ 4.396	0.670	0.406	0.201 ∼ 1.722
Depth of invasion (≤2/3, >2/3)	1.358	0.403	0.663 ∼ 2.783			
HR HPV infection (Negative, Positive)	1.501	0.347	0.643 ∼ 3.502			
Tumor size	1.243	0.132	0.936 ∼ 1.652			
LVSI (Negative, Positive)	0.797	0.538	0.387 ∼ 1.643			
Age	0.998	0.894	0.967 ∼ 1.030			
Histology (Squamous, Adenocarcinoma)	0.927	0.961	0.412 ∼ 2.240			
Menopause (Yes, No)	1.426	0.333	0.696 ∼ 2.921			

*HR* hazard ratio

### Methylation status of the *MEG3* promoter in cervical cancer tissues

**Fig. 3:**
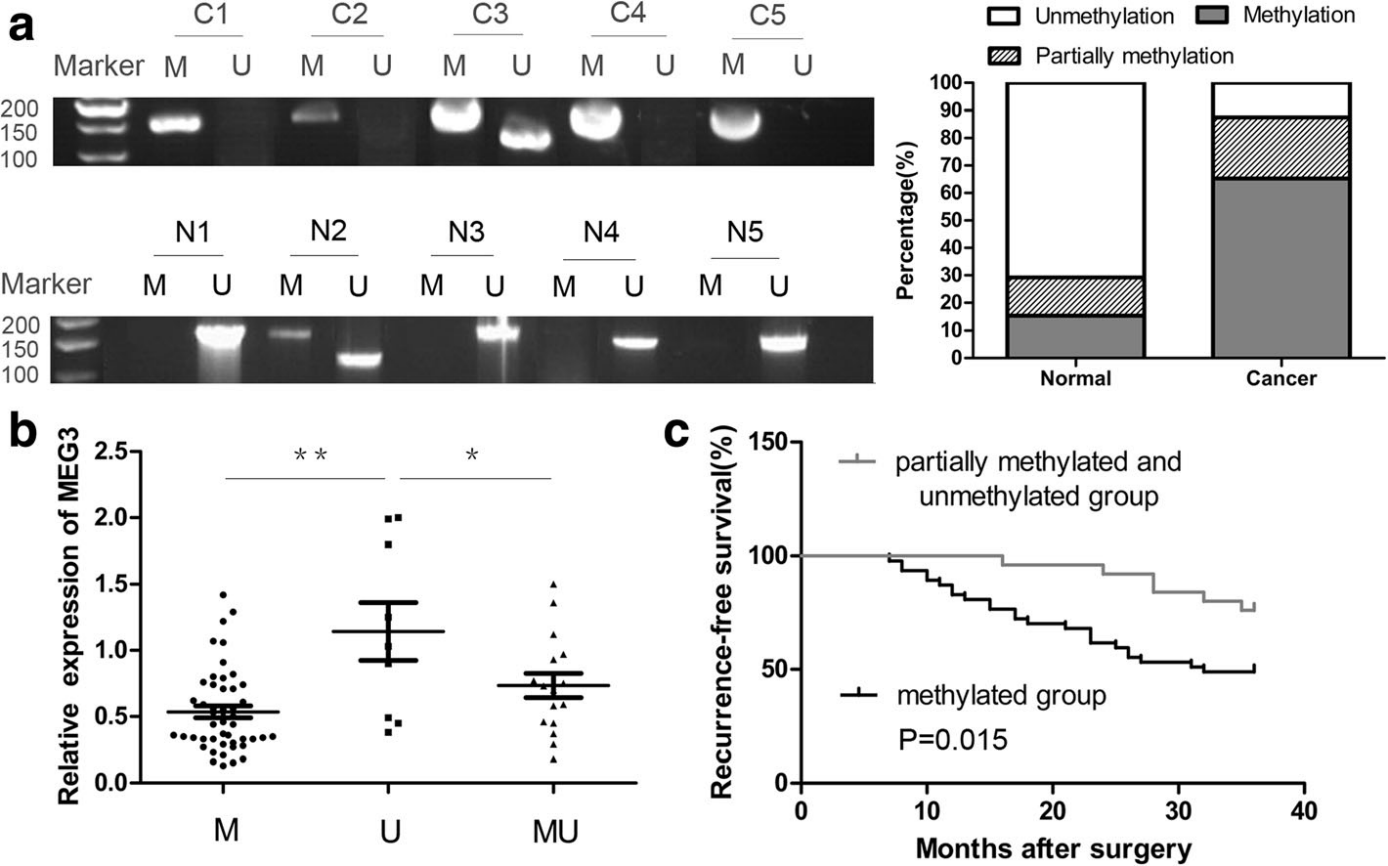
Methylation status of the *MEG3* promoter in cervival cancer. **a** Methylation status of *MEG3* promoter in cervival cancer tissues (C1-C5 as example) and its corresponding normal tissues (N1-N5 as example) was tested by MSP. The methylated pattern (M) of *MEG3* was 160 bp and the unmethylated pattern (U) was 120 bp. **b** Patients were classified into methylation group (M), partially methylated group (MU) and unmethylated group (U) based on the methylation levels of *MEG3* promoter. *MEG3* expression level was associated with the methylation level of *MEG3* promoter. **c** Hypermethylation of *MEG3* promoter indicated poor recurrence-free survival. ^*^ *P* < 0.05, ^**^ *P* < 0.01

The methylation status of the *MEG3* promoter was assessed by MSP. The unmethylated pattern (U), partially methylated pattern (M and U), and methylated pattern (M) were observed in 12.5%, 22.2%, and 65.3% of cervical cancer tissues, respectively. In comparison, the unmethylated pattern (70.8%) was more common than the methylated (13.9%) or partially methylated patterns (15.3%) in corresponding normal tissues (Fig. 3a, Table 2). The difference in promoter methylation status between cervical cancer tissues and corresponding normal tissues was statistically significant (*P* < 0.001, Table 2). More importantly, there was a positive correlation between cervical cancer and promoter hypermethylation (*R* = 0.592, Table 2). *MEG3* methylation was a risk factor for cervical cancer (OR = 17, Table 2).

**Table 2:** Correlation between cervical cancer and methylation status of *MEG3* promoter

Tissues	Methylation status		χ^2^	P	R	OR (95% CI)
	M, MU	U				
Cervical cancer	63	9	50.4	<0.001	0.592	17.000 (7.167–40.324)
Normal	21	51				

*OR* odds ratio

To better understand the role of methylation, we also analyzed the correlation between *MEG3* expression and promoter methylation level in cervical cancer tissues. As shown in Fig. 3b, expression of *MEG3* was significantly higher in the unmethylated group than in the methylated or partially methylated groups.

Furthermore, HR-HPV infection and lymph node metastasis were significantly correlated with *MEG3* methylation (Table 3). The Chi-square test also indicated that *MEG3* methylation was a risk factor for HR-HPV infection and lymph node metastasis (Table 3). However, *MEG3* methylation was not significantly correlated with age, menopause, histology, differentiation, FIGO stage, tumor size, depth of invasion, or LVSI in cervical cancer (Table 3). Moreover, Kaplan–Meier analysis showed that the promoter methylation level of *MEG3* was significantly correlated with recurrence-free survival time: patients in the methylated group had significantly shorter recurrence-free survival times than those in the partially methylated or unmethylated groups (*P* = 0.015, Fig. 3c).

**Table 3:** Relationship between *MEG3* methylation and clinical pathological characteristics in patients with cervical cancer

Clinical pathological characteristics	P	R	OR (95% CI)
HR HPV infection (Positive, Negative)	0.013	0.291	3.830 (1.279–11.467)
Lymph nodes metastasis (Positive, Negative)	0.025	0.264	3.520 (1.131–10.953)
FIGO stage (II, I)	0.056		
Depth of invasion (>2/3, ≤2/3)	0.106		
Differentiation (Well/Moderately, Poorly)	0.741		
Tumor size (>4 cm, ≤4 cm)	0.703		
LVSI (Positive, Negative)	0.675		
Age (>50, ≤50)	0.371		
Histology (Squamous, Adenocarcinoma)	0.899		
Menopause (Yes, No)	0.658		

*OR* odds ratio

### Epigenetic regulation of *MEG3* in cervical cancer cells

**Fig. 4:**
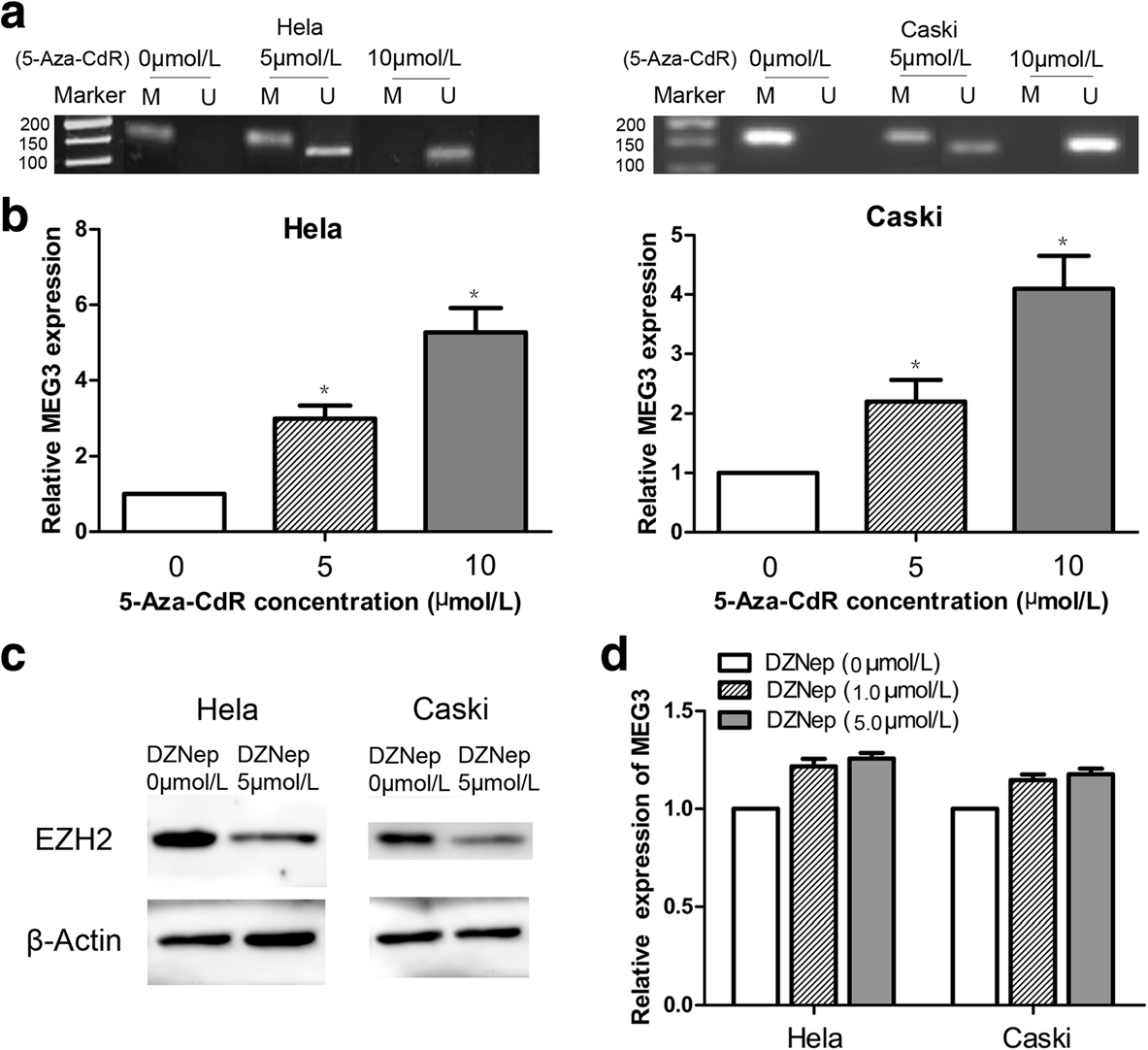
The effects of 5-aza-CdR and DZNeP on *MEG3* expression. **a** *MEG3* promoter was hypermethylation in HeLa and CaSki cells (0 μmol/L). *MEG3* promoter could be demethylation by different concentrations of 5-aza-CdR (5 and 10 μmol/L) in HeLa and CaSki cells. **b** *MEG3* was re-expressed in the HeLa and CaSki cells treated with different concentrations of 5-aza-CdR (5 and 10 μmol/L), compared to cells of the control group (0 μmol/L 5-aza-CdR). **c** EZH2 protein levels in cells treated with DZNep (0, 5 μmol/L) were examined by western blot **d** *MEG3* expression was analyzed by RT-qPCR in cells treated with DZNep (0, 1, 5 μmol/L) for 5 days and there was no significant statistical difference among the groups

As the MSP assay showed, the promoter of *MEG3* was hypermethylated in HeLa and CaSki cells (0 μmol/L, Fig. 4a). To investigate the role of promoter methylation in regulation of *MEG3* expression in cervical cancer cells, we examined the effect of 5-aza-CdR on promoter methylation levels and *MEG3* expression. Compared with the control treatment, 5-aza-CdR treatment significantly decreased the promoter methylation level in HeLa and CaSki cells (Fig. 4a). Furthermore, qRT-PCR analysis indicated that expression of *MEG3* was significantly higher in the 5-aza-CdR treatment group than in the control group (Fig. 4b, *P* < 0.05).

In addition, we also examined the effects of DZNep treatment based on HeLa and CaSki cells. We first confirmed the significant reduction of EZH2 protein levels (Fig. 4c). However, no significant change in *MEG3* expression levels was observed after treatment with DZNep (Fig. 4d).

### Methylation status of the *MEG3* promoter influenced proliferation of cervical cancer cells

**Fig. 5:**
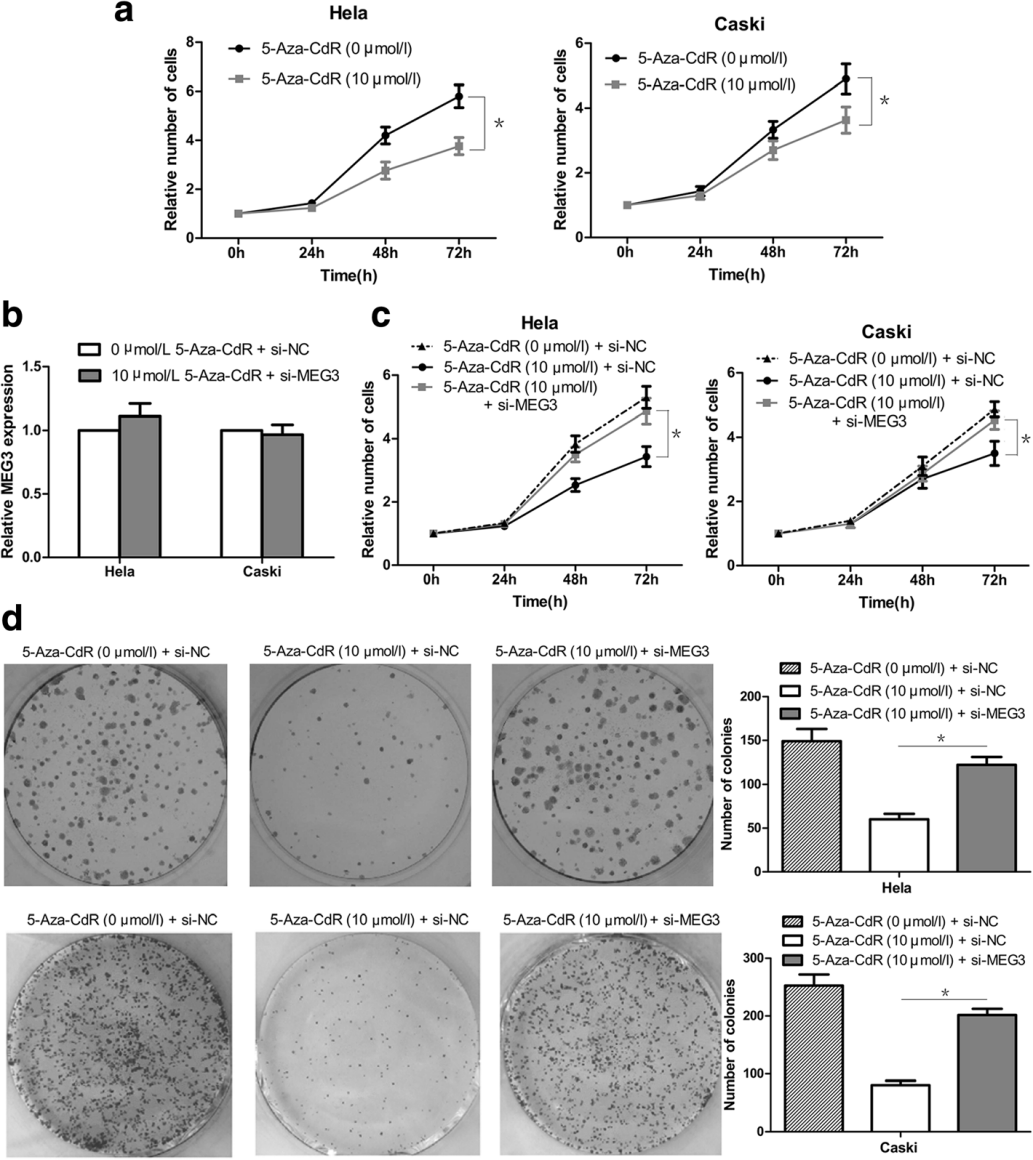
The effects of 5-aza-CdR on cervical cancer cells proliferation in vitro. **a** 5-aza-CdR dramatically decreased the proliferation ability of HeLa and CaSki cells by CCK-8 assay. **b** si-*MEG3* was just canceled out the promoting effect of 5-aza-CdR on *MEG3* expression. **c** and **d** si-*MEG3* could partly reverse the inhibitory effect of 5-aza-CdR on proliferation of HeLa and CaSki cells, as shown by CCK-8 assays and Colony formation assays. ^*^ *P* < 0.05

As shown by the CCK-8 assay, 5-aza-CdR significantly decreased the proliferation ability of HeLa and CaSki cells, compared with control cells (Fig. 5a, *P* < 0.05). According to the colony formation assay, 5-Aza-CdR (10 μmol/L 5-Aza-CdR + si-NC group) also resulted in a notable decrease in the number of colonies, as compared with the control groups (0 μmol/L 5-Aza-CdR + si-NC group) (Fig. 5d *P* < 0.05). To prevent excessive use of si-*MEG3*, qRT-PCR confirmed that there was no significant difference in *MEG3* expression between the 5-aza-CdR (10 μmol/L) + si-*MEG3* and the si-NC groups (Fig. 5b). The efficiency of si-*MEG3* was tested in our previous study. Furthermore, the CCK-8 and colony formation assays indicated that the proliferation ability of HeLa and CaSki cells in the 5-aza-CdR (10 μmol/L) + si-*MEG3* group was significantly higher than that in the 5-aza-CdR (10 μmol/L) + si-NC group (Fig. 5c, d, *P* < 0.05).

## Discussion

Our previous study showed that *MEG3* expression levels are related to HR-HPV infection, tumor size, FIGO stage, and lymph node metastasis in patients with cervical cancer [13]. However, the value of *MEG3* in clinical practice is generally unknown. In the present study, we identified *MEG3* as a potential marker for lymph node metastasis. Then we tested the ROC model using data from 108 other patients with cervical cancer from our previous study. The results confirmed that the *MEG3* expression cut-off value that we set previously for prediction of lymph node metastasis was still effective, supporting our previous results. The presence or absence of lymph node metastasis is a crucial factor in therapy decision-making for patients with cervical cancer and our study found that *MEG3* is a good candidate to predict it. That indicates that *MEG3* shows excellent potential value for clinical work, especially for cervical cancer, in which biopsy is routine before therapy.

The lack of valid prognostic prediction models makes it difficult to apply individualized therapy to patients with cervical cancer. Developing a method for identifying patients at high risk of treatment failure is important. For these high-risk patients, modified therapies such as neoadjuvant radiation or chemotherapy could potentially be applied to improve patient survival. Although extensive effort has been devoted to understanding the prognostic value of *MEG3* in human tumors [8, 10, 11], little was known about its value in cervical cancer until now. In the present study, we evaluated the prognostic value of *MEG3* using Kaplan–Meier and Cox regression analyses. Kaplan–Meier analysis of patients with cervical cancer indicated that *MEG3* expression level was associated with recurrence-free and overall survival. Furthermore, *MEG3* was confirmed to be an independent prognostic marker for recurrence-free survival by multivariate analysis. Therefore, *MEG3* may represent a novel indicator of prognosis in cervical cancer, helping to identify high-risk patients before treatment.

Emerging results indicate that epigenetic aberrations, especially DNA methylation, regulate tumor growth by silencing tumor suppressor genes [16, 17]. The promoter region as well as the intergenic germ line-derived differentially methylated region (IG-DMR) of *MEG3* is rich in CpG dinucleotides [18]. Thus, many studies have reported that promoter methylation plays an important role in loss of *MEG3* expression in tumors [8, 11, 19]. In this study, we tested the hypothesis that promoter hypermethylation may be able to reduce *MEG3* levels in cervical cancer, by evaluating the methylation level of the *MEG3* promoter and its effects on *MEG3* expression. Similar to other findings, low *MEG3* expression was shown to be related to hypermethylation of the *MEG3* promoter in cervical cancer tissues.

Further, we endeavored to reveal the role of *MEG3* methylation in the progress of cervical cancer. We found that *MEG3* methylation is not only a risk factor for cervical cancer, but also for HR-HPV infection and lymph node metastasis. More importantly, patients with hypermethylation of the *MEG3* promoter showed poor recurrence-free survival. Collectively, these results indicate the importance of *MEG3* methylation in cervical cancer progression. Interestingly, we noted that the role of *MEG3* methylation was consistent with that of *MEG3* expression in cervical cancer, and the clinical characteristics of patients in the methylated group were similar to those of patients in the *MEG3*-Low group, according to our previous study. These results provide strong evidence for the relevance of *MEG3* inactivation and promoter hypermethylation.

In addition, we showed, using cell-based experiments, that promoter methylation and *MEG3* expression are correlated. We revealed that the *MEG3* promoter is hypermethylated in cervical cancer cells, and that demethylation of the promoter resulted in re-expression of *MEG3*. More importantly, as the promoter methylation level decreased, the expression of *MEG3* increased in cervical cancer cells, which indicated that loss of *MEG3* expression in cervical cancer cells was the result of promoter hypermethylation, at least in part. Moreover, our results indicated that a decrease in the methylation level resulted in not only re-expression of *MEG3*, but also reduction of the proliferation potential in cervical cancer cells. Furthermore, repression of *MEG3* re-expression could partly reverse the inhibitory effect of 5-aza-CdR on proliferation of HeLa and CaSki cells, revealing that *MEG3* re-expression may play a crucial role in 5-aza-CdR functions. Based on the above results, we have a clear indication that *MEG3* re-expression owing to DNA demethylation could inhibit proliferation of cervical cancer cells. The results also indicate that inactivation of *MEG3* via promoter hypermethylation plays an important role in regulation of cervical cancer cell proliferation. In addition, it confirms the results of our previous study, that down-regulation of *MEG3* could suppress the proliferation of cervical cancer cells in another way. In summary, we propose that the following process may occur in cervical cancer cells: promoter hypermethylation → inactivation of *MEG3* → malignant cell proliferation.

In addition to DNA methylation, we are also interested in the effect of histone methylation on *MEG3* expression. DZNep has been shown to reduce expression of EZH2, inhibit methylation of H3K27, and affect histone methylation [20]. However, DZNep did not significantly affect *MEG3* expression in cervical cancer cells, implying that histone methylation may not be a major mechanism of *MEG3* inactivation. However, this is just the first step in study of histone methylation of *MEG3* and much remains to be done.

## Conclusions

*MEG3* may be a useful diagnostic tool and prognostic marker for cervical cancer, and its inactivation in cervical cancer may be due to promoter hypermethylation. *MEG3* shows potential for use in diagnostic applications and therapeutic interventions in cervical cancer.

## Additional file

Additional file 1: Table S1. Patients’ clinico-pathological characteristics. (DOC 40 kb)

## Abbreviations

5-Aza-CdR: 5-aza-2-deoxy-cytidine
AUC: The areas under the ROC curve
CCK-8: Cell Counting Kit-8
lncRNA: Long noncoding RNA
LVSI: Lymphatic vascular space invasion
MEG3: Maternally expressed gene 3
MSP: Methylation specific PCR
ROC: Receiver operating characteristic curve
