# Supplementary material for: Downregulation of long noncoding RNA MEG3 is associated with poor prognosis and promoter hypermethylation in cervical cancer
Source: J Exp Clin Cancer Res. 2017 Jan 5;36:5. doi: 10.1186/s13046-016-0472-2 (PMC5216566; doi:10.1186/s13046-016-0472-2)
Supplement: Additional file 1: Table S1. — Patients’ clinico-pathological characteristics. (DOC 40 kb) [file 13046_2016_472_MOESM1_ESM.doc]

Table S1 Patients’ clinico-pathological characteristics

| Clinico-pathologic feature | N (%) |
| --- | --- |
| Age |  |
| ≤50 | 35(48.6%) |
| >50 | 37(51.4%) |
| Menopause |  |
| Yes | 33(45.8%) |
| No | 39(54.2%) |
| Histology |  |
| Squamous cell cancer | 51(70.8%) |
| Adenocarcinoma | 16(22.1%) |
| Other | 5(11.1%) |
| Differentiation |  |
| Well to moderately | 44(61.1%) |
| Poorly | 28(38.9%) |
| Tumor size |  |
| <4cm | 40(55.6%) |
| ≥4cm | 32(44.4%) |
| Depth of invasion |  |
| ≤2/3 | 40(55.6%) |
| >2/3 | 32(44.4%) |
| Lymphatic vascular space invasion |  |
| Negative | 35(48.6%) |
| Positive | 37(51.3%) |
| Lymph node metastasis |  |
| Negative | 43(59.7%) |
| Positive | 29(40.3%) |
| HR‑HPV infection |  |
| Negative | 18(25.0%) |
| Positive | 54(75.0%) |
| FIGO stage |  |
| I | 46(63.9%) |
| II | 26(36.1%) |
